# Genome-wide association study of platelet aggregation in African Americans

**DOI:** 10.1186/s12863-015-0217-9

**Published:** 2015-05-30

**Authors:** Rehan Qayyum, Lewis C. Becker, Diane M. Becker, Nauder Faraday, Lisa R. Yanek, Suzanne M. Leal, Chad Shaw, Rasika Mathias, Bhoom Suktitipat, Paul F. Bray

**Affiliations:** The GeneSTAR Research Center, Department of Medicine, Johns Hopkins University School of Medicine, Baltimore, Maryland 21287 USA; Department of Medicine, University of Tennessee College of Medicine, Chattanooga, Tennessee 37403 USA; Department of Anesthesiology and Critical Care Medicine, Johns Hopkins University School of Medicine, Baltimore, Maryland 21287 USA; Molecular and Human Genetics, Baylor College of Medicine, Houston, Texas 77030 USA; Integrative Computational BioScience Center, Department of Biochemistry, Faculty of Medicine, Siriraj Hospital, Mahidol University, Bangkok,, 10700 Thailand; Department of Medicine, Thomas Jefferson University, Jefferson Medical College, Philadelphia, Pennsylvania 19107 USA

**Keywords:** Platelet aggregation, *PEAR1*, *BMPR1A*, Genome-wide association study

## Abstract

**Background:**

We have previously shown that platelet aggregation has higher heritability in African Americans than European Americans. However, a genome-wide association study (GWAS) of platelet aggregation in African Americans has not been reported. We measured platelet aggregation in response to arachidonic acid, ADP, collagen, or epinephrine by optical aggregometry. The discovery cohort was 825 African Americans from the GeneSTAR study. Two replication cohorts were used: 119 African Americans from the Platelet Genes and Physiology Study and 1221 European Americans from GeneSTAR. Genotyping was conducted with Illumina 1 M arrays. For each cohort, age- and sex-adjusted linear mixed models were used to test for association between each SNP and each phenotype under an additive model.

**Results:**

Six SNPs were significantly associated with platelet aggregation (P < 5×10^−8^) in the discovery sample. Of these, three SNPs in three different loci were confirmed: 1) rs12041331, in *PEAR1* (platelet endothelial aggregation receptor 1), replicated in both African and European Americans for collagen- and epinephrine-induced aggregation, and in European Americans for ADP-induced aggregation; 2) rs11202221, in *BMPR1A* (bone morphogenetic protein receptor type1A), replicated in African Americans for ADP-induced aggregation; and 3) rs6566765 replicated in European Americans for ADP-induced aggregation. The rs11202221 and rs6566765 associations with agonist-induced platelet aggregation are novel.

**Conclusions:**

In this first GWAS of agonist-induced platelet aggregation in African Americans, we discovered and replicated, novel associations of two variants with ADP-induced aggregation, and confirmed the association of a *PEAR1* variant with multi-agonist-induced aggregation. Further study of these genes may provide novel insights into platelet biology.

**Electronic supplementary material:**

The online version of this article (doi:10.1186/s12863-015-0217-9) contains supplementary material, which is available to authorized users.

## Background

Human blood platelets play an essential role in normal hemostasis and in pathologic thrombosis, in particular arterial thrombosis [1]. There is accumulating evidence that platelets also participate in the development, progression, and manifestations of atherosclerotic diseases [2]. Studies of ex vivo agonist-induced platelet aggregation have shown large and reproducible variations among individuals [3]. In patients receiving anti-platelet therapy for secondary cardiovascular prevention, greater platelet aggregability ex vivo is associated with increased risk of cardiovascular disease events [4, 5]. Several cardiovascular risk factors are known to affect platelet aggregation in healthy individuals including age, sex, obesity, and the presence of metabolic syndrome [6–10]. The majority of this variation in platelet aggregation is heritable. Using a family study design, we have previously reported that greater than 70 % of variation in platelet aggregation in African Americans and almost 60 % of variation in European Americans is heritable [11].

Several candidate gene studies have examined the association of genetic variants in specific genes with platelet aggregation with inconsistent results [12]. A genome-wide association study in European Americans identified seven loci associated with agonist-induced platelet aggregation [13]. However, a genome-wide association study of platelet aggregation in African Americans has not yet been reported. The genetic variants reported to date explain only a small fraction of the heritability in platelet aggregation, providing an opportunity for new studies to discover additional genetic variants of importance. Moreover, African Americans have higher heritability of platelet aggregation than European Americans and additional genetic variants may contribute to this difference [11]. Because of the different allele frequencies and linkage disequilibrium patterns in populations of European and African ancestry, we anticipated that we might discover new genetic loci associated with platelet aggregation in an African American population compared to European Americans [14]. Therefore, we performed a genome-wide association study (GWAS) in African Americans to identify genetic variants that determine agonist-induced platelet aggregation and replicated our findings in independent samples of African Americans and European Americans [4, 5].

## Results

The discovery sample from GeneSTAR consisted of 825 African Americans. The replication samples consisted of 119 African Americans in PGAP and 1221 European Americans in GeneSTAR (Table 1). Participants from the GeneSTAR cohorts were older with greater percentages of hypertensives and smokers. They also had higher platelet count and fibrinogen levels. Overall, 802,881 genotyped SNPs passed our quality control criteria in all studies and were included in analyses. Table 2 shows the gene variants that were significantly associated with agonist-induced platelet aggregation at the GWAS level in African American GeneSTAR participants. The quantile-quantile plots are presented in Additional file 1: Fig. S1 with inflation of test statistics (lambda range 1.04 to 1.08).

**Table 1:** Study population characteristics

Characteristics	GeneSTAR (AA) (N = 825)	PGAP (AA) (N = 119)	GeneSTAR (EA) (N = 1221)
Age, years	45 (12)	35 (9)	44 (13)
Female	62 %	72 %	55 %
Hypertension	39 %	8 %	26 %
Smoker	30 %	14 %	23 %
Body Mass Index, Kg/m^2^	32 (8)	29 (6)	28 (6)
Fibrinogen, mg/dL	375 (111)	349 (101)	374 (111)
Platelet Count, 10^9^/L	266 (68)	236 (53)	261 (62)
Mean Platelet Volume, 10^−15^ L	8 (1)	7 (1)	7 (1)
Von Willebrand Factor, %	86 (53)	87 (38)	87 (58)

Data is presented as mean (standard deviation) unless noted otherwise

*Abbreviations*: *EA* European Americans, *AA* African Americans

Population Characteristics of the GeneSTAR (Genetic Study of Atherosclerosis Risk) and PGAP (Platelets Genes and Physiology) cohorts

**Table 2:** Genome-wide association study results

			GeneSTAR (AA)			PGAP			GeneSTAR (EA)		
SNP_CA	Position	Gene	β(SE)	P-value	MAF	β(SE)	P-value	MAF	β(SE)	P-value	MAF
**Epinephrine 2 μM**											
rs12041331_A	1:155136338	PEAR1	−0.93 (0.13)	2.82 × 10-^12^	29.2 %	−0.19 (0.05)	4.64 × 10^−3^	32.8 %	−0.47 (0.18)	4.47 × 10^−3^	9.1 %
**Collagen 2 μg/mL**											
rs12041331_A	1:155136338	PEAR1	−0.89 (0.13)	2.74 × 10^−11^	35.8 %	−1.55 (0.59)	9.0 × 10^−3^	32.1 %	−0.47 (0.18)	0.01	9.1 %
**ADP 2 μM**											
rs12041331_A	1:155136338	PEAR1	−9.2 (1.56)	5.8 × 10^−9^	35.8 %	−3.65 (2.41)	0.13	33.2 %	−8.52 (1.87)	6.08 × 10^−6^	9.1 %
rs11924165_T	3:127391893	ALDH1L1-AS2	9.72 (1.68)	1.17 × 10^−8^	16.9 %	−5.10 (2.86)	0.07	20.0 %	-	-	-*
rs10883735_T	10:104298436	SUFU	−13.9 (2.45)	2.18 × 10^−8^	5.1 %	1.72 (4.28)	0.69	8.3 %	1.36 (2.45)	0.48	8.1 %
rs11202221_G	10:88592294	BMPR1A	−12.72 (2.30)	4.8 × 10^−8^	7.3 %	−16.04 (4.06)	7.71 × 10^−5^	9.1 %	0.60 (1.50)	0.69	20.5 %
**ADP 10 μM**											
rs12041331_A	1:155136338	PEAR1	−5.63 (0.88)	3.2 × 10-^10^	35.8 %	−3.65 (2.41)	0.13	32.1 %	−2.57 (1.36)	0.06	9.1 %
rs6566765_T	18:69569776		4.72 (0.84)	3.59 × 10^−8^	39.7 %	1.78 (2.49)	0.34	39.3 %	2.01 (0.62)	1.3 × 10^−3^	35.1 %
rs9889955_G	17:69072972	SDK2	−6.42 (1.15)	4.14 × 10^−8^	23.5 %	−0.47 (2.71)	0.86	25.9 %	−0.32 (0.74)	0.66	39.3 %†

*Abbreviations:* *SNP* single nucleotide polymorphism, *CA* coded allele, *β* regression coefficient, *SE* standard error, *GeneSTAR* Genetic Study of Atherosclerosis, *PGAP* Platelet Gene and Physiology, *MAF* minor allele frequency, *ADP* adenosine diphosphate, *AA* African Americans, *EA* European Americans

Note: In PGAP the epinephrine concentration of 1.5 uM, ADP concentration of 4 uM, and collagen concentration of 2.5 ug/mL were used

* This SNP is monomorphic in population of European descent. Another SNP, rs7611945_A, 231,689 bp upstream of rs11924165 was statistically significant (β(SE) = −4.68 (1.32); p = 4.25 × 10^−4^; MAF = 20 %) after adjusting for the number of SNPs in between (N = 97) or the number of LD blocks (N = 20)

† Another variant in SDK2 gene, rs11869008_G, located 140,506 bp downstream of rs9889955 was statistically significant (β(SE) = −2.79 (0.84); p = 9.66 × 10^−4^; MAF = 19.7 %) after adjusting for the number of LD blocks in YRI (N = 25) but not after adjusting for the number of genotyped SNPs (N = 63)

Genome-wide association study results of platelet aggregation in discovery cohort of African Americans and replication cohorts of African Americans and European Americans

**Fig. 1:**
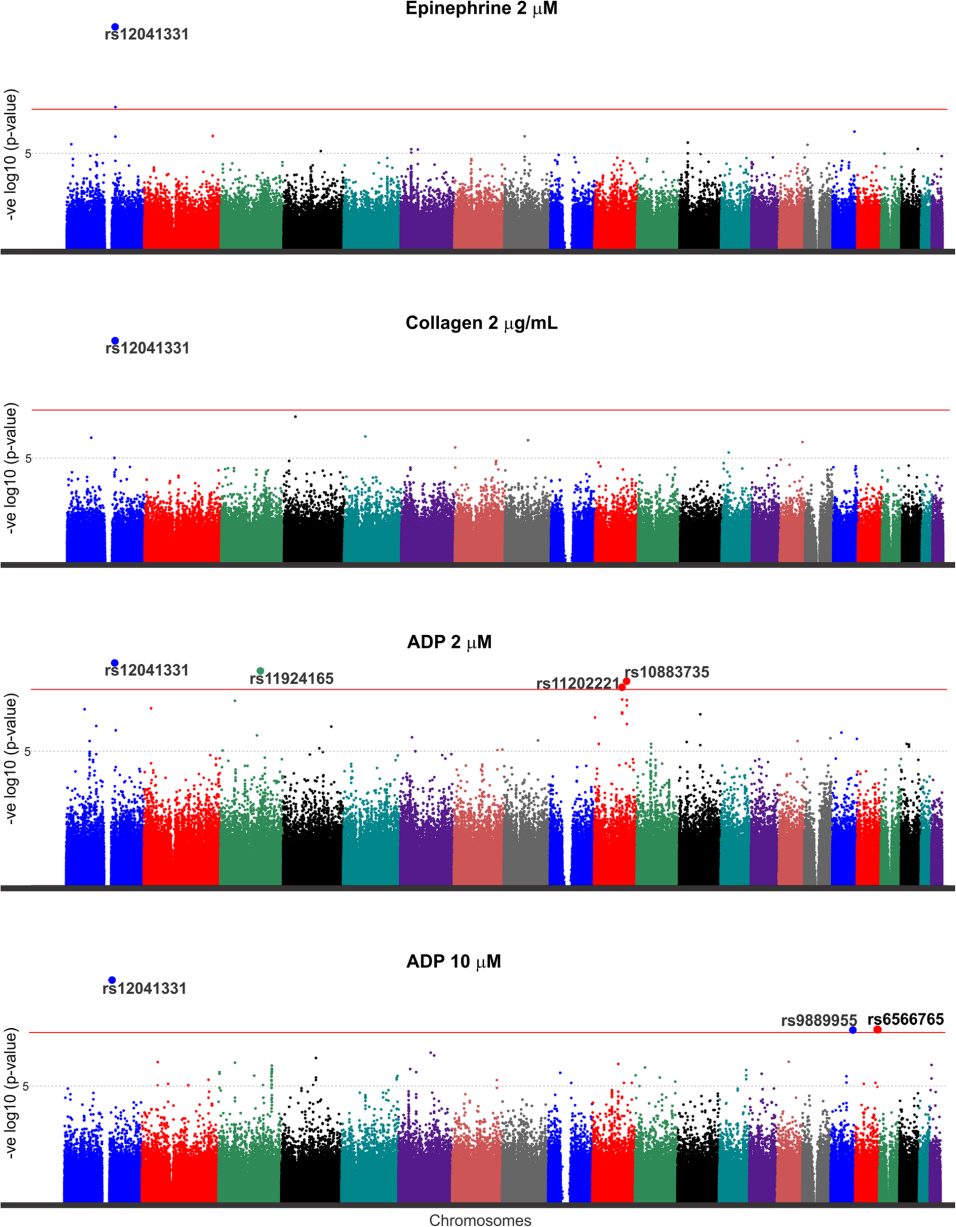
Manhattan plot of the genome-wide association results of agonist-mediated platelet aggregation. The y-axis represents the negative logarithm (base 10) of p-values and the x-axis represents chromosomes with positions of genetic variants. The horizontal red line represents the genome-wide significance threshold. Results of arachidonic acid-mediated platelet aggregation are not shown here as no genetic variant crossed genome-wide significance threshold

### Epinephrine-induced platelet aggregation

The A-allele of rs12041331, located in the first intron of *PEAR1*, was associated with decreased platelet aggregation to 2 μM epinephrine (p = 8.34 × 10^−12^) (Fig. 1 and Additional file 1 Table S1). The association of this SNP has been previously reported by our group and the Framingham Heart Study with platelet aggregation phenotypes in both European Americans and African Americans [13, 15]. Both replication cohorts validated the association of this SNP with epinephrine-induced platelet aggregation (African Americans: p = 4.6 × 10^−3^; European Americans: p = 4.47 × 10^−3^) with a similar direction of effect. Suggestive association was noted with SNPs at 6 loci but none were significant in the replication samples (Additional file 1: Table S2).

### Collagen-induced platelet aggregation

The *PEAR1* SNP rs12041331 was also associated with platelet aggregation to 2 μg/mL collagen (Fig. 1; p = 2.7 × 10^−11^). This association was also present in both replication cohorts (African American: p = 9.0 × 10^−3^; European American: p = 0.01) although p-values did not cross Bonferroni-adjusted p-value threshold. The minor allele, A, was associated with lesser platelet aggregation in all cohorts. In addition, 6 SNPs had suggestive significant association with aggregation to with 2 μg/mL collagen (Additional file 1: Table S3).

### ADP-induced platelet aggregation

**Fig. 2:**
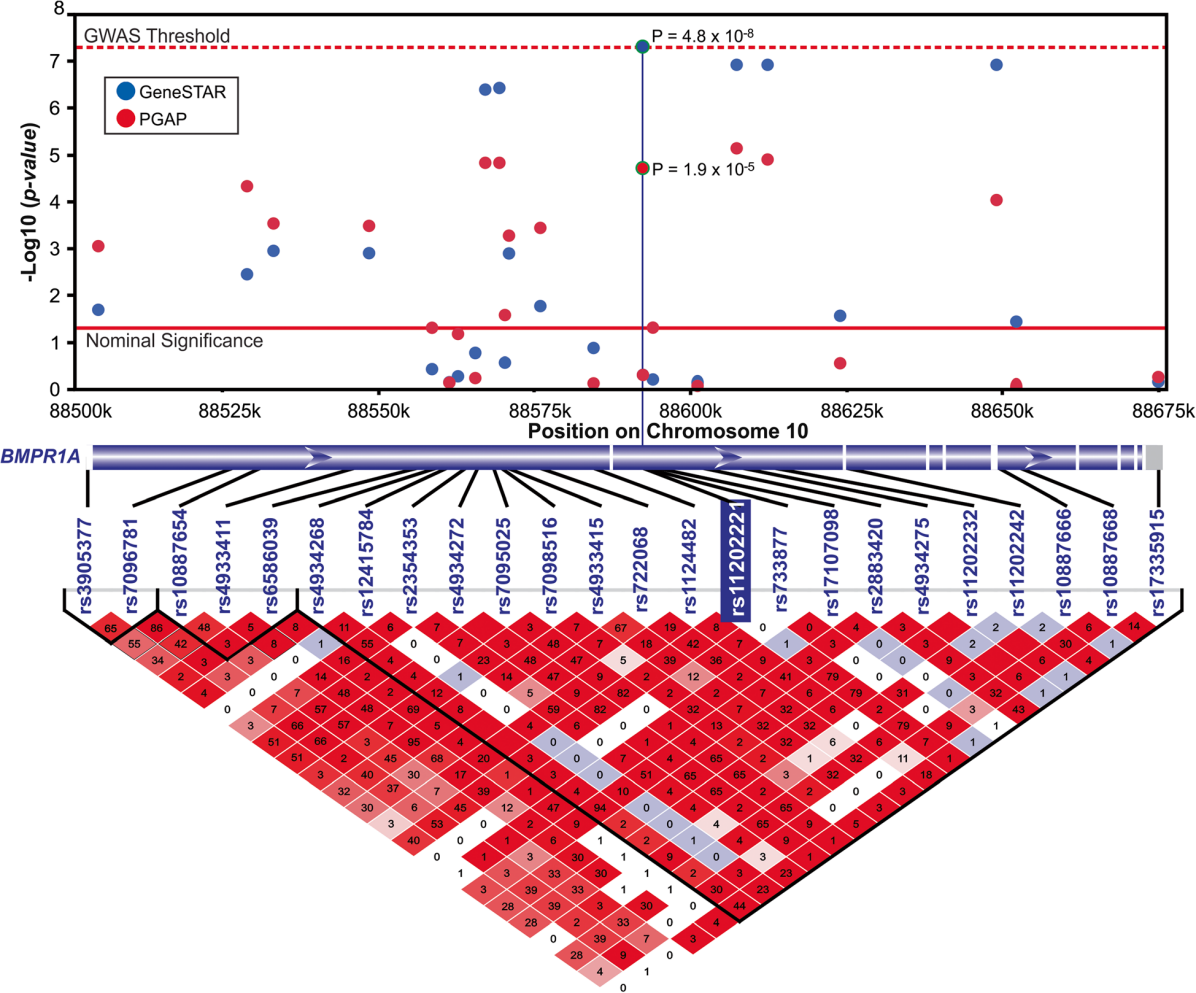
Upper half: Results of association between genetic variants in BMPR1A gene and ADP 2 μM in the GeneSTAR African American cohort and PGAP with the vertical bar highlighting the lead genetic variant in the region. The y-axis represents the negative logarithm (base 10) of p-values and the x-axis represent the base-pair position of genetic variants on chromosome 10. Lower half: Linkage disequilibrium plot of the BMPR1A region in the GeneSTAR African American cohort. Lead genetic variant in the region is highlighted with a blue background

The *PEAR1* SNP rs12041331 was also significantly associated with platelet aggregation after stimulation with both doses of ADP (p = 5.8 × 10^−9^ for 2 μM ADP and p = 3.2 × 10^−10^ for 10 μM ADP) (Fig. 1). Three additional SNPs were significantly associated with platelet aggregation after stimulation with 2 μM ADP. One SNP, rs11924165, was in the non-protein coding region of *ALDH1L1-AS2* (aldehyde dehydrogenase 1 family, member L1-antisense RNA 2); the minor allele was associated with increased platelet aggregation (P = 1.17 × 10^−8^). The second SNP, rs10883735, was located in the 2^nd^ intron of *SUFU* gene (p = 2.2 × 10^−8^). The third SNP, rs11202221, was located in the 2nd intron of the *BMPR1A* gene (p = 4.8 × 10^−8^). The minor allele of this SNP was associated with decreased platelet aggregation (Additional file 1: Table S1 and S4). Five additional SNPs in *BMPR1A* had suggestive associations with p-values < 5 × 10^−7^ (Fig. 2; Additional file 1: Table S5); all five SNPs were in strong LD with the lead SNP, rs11202221 *of BMPR1A* (Fig. 2).

Two additional SNPs were significantly associated with platelet aggregation after stimulation with 10 μM ADP (Table 2). One SNP, rs6566765, was located in an intergenic region with no known protein-coding gene within the flanking 250 kb and its minor allele was associated with increased platelet aggregation (Additional file 1: Table S1 and S4). Another SNP, rs9889955, was located in the first intron of *SDK2.*

Of the six SNPs associated with ADP-induced platelet aggregation in the discovery sample, only the G allele of the rs11202221 in *BMPR1A* was associated with decreased ADP-induced platelet aggregation in the African American replication sample (p = 9.17 × 10^−5^; Table 2). Furthermore, the 5 SNPs with suggestive significance in *BMPR1A* in the discovery sample were also significant in the African American replication sample (Fig. 2; all p-values < 9 × 10^−5^). However, neither rs11202221 nor any nearby SNP was associated with ADP-induced aggregation in the European American replication cohort, likely due to differences in linkage disequilibrium between the two populations (Additional file 1: Fig. S3).

The A allele of rs12041331 in *PEAR1* was not significantly associated with platelet aggregation in the PGAP replication sample, although the direction of effect was similar for both concentrations of ADP (p = 0.14). On the other hand, this SNP was associated with decreased platelet aggregation after stimulation with 2 μM ADP in the European American cohort and had a similar direction of effect (β[SE] = −9.2[1.56], p = 5.8 × 10^−9^). The T allele of the intergenic variant, rs6566765, was associated with increased platelet aggregation to 10 μM ADP in the European American cohort (β[SE] = 2.01[0.62]; p = 1.3 × 10^−3^) but not in the African American replication cohort. When ADP-mediated platelet aggregation with both doses was examined across the genotypes of rs11202221 and rs6566765, we found that increasing allele dose (G-allele for rs11202221 and C-allele for rs6566765) was associated with decreased platelet aggregation (Additional file 1: Table S4). The rs9889955 variant in *SDK2* was not associated with platelet aggregation in either of the two replication cohorts.

Suggestive associations between platelet aggregation induced by 2 μM ADP and 10 μM ADP were noted for SNPs at 16 loci and 23 loci, respectively, but only 1 of these SNPs was replicated (Additional file 1: Tables S5 and S6). The minor allele of the replicated variant, rs750693 in *FRMPD1*, was also associated with an increase in platelet aggregation in response to 10 uM ADP in the European American replication sample.

### Arachidonic acid-induced platelet aggregation

We did not find any locus that crossed GWAS threshold for arachidonic acid-mediated platelet aggregation in the discovery sample. We found 10 loci that had suggestive association with arachidonic acid-induced platelet aggregation (Additional file 1: Table S7), but none were nominally associated with arachidonic acid-induced aggregation in the replication samples.

## Discussion

Lower coronary artery disease survival rates have been observed in African Americans even after clinical, demographic and socioeconomic variables are considered [16, 17], suggesting there are undiscovered factors accounting for this racial difference. We and others have shown there is a strong genetic component to coronary artery disease and platelet reactivity [11, 18–20], but there is a paucity of data about responsible genetic mechanisms. Thus, the African American participants in the GeneSTAR and PGAP studies represent unique and valuable resources for discovering novel genetic variants associated with platelet aggregation, a central process in acute coronary syndromes. We used the large GeneSTAR African American cohort as the GWAS discovery sample, and separate PGAP and GeneSTAR European Americans as replication cohorts. The major findings were: 1) identification of 3 replicated variants associated with platelet aggregation in the discovery sample, 2) rs12041331 in *PEAR1* (previously reported genetic variant in European Americans and African Americans), was associated with collagen, epinephrine and ADP aggregation in both the discovery and replication cohorts, 3) rs11202221 in *BMPR1A* was associated with ADP aggregation in both the discovery and the African American replication cohort, and 4) rs6566765 was associated with ADP aggregation in the African American discovery and European American replication cohort. Since African Americans are under-represented in most clinical studies of coronary artery disease (CAD), it will be important to consider these established and novel genetic variants in this racial group.

Despite modest sample sizes of the discovery and replication cohorts, we were able to identify and validate three loci associated with platelet aggregation. The finding of significant loci in modestly sized cohorts is not typical of most GWAS studies. We had large effect sizes for these loci, probably because platelet aggregation is more physiologically defined than most clinical phenotypes and represents a biological process, not a disease outcome. The large effect sizes are likely due to the relatively large percentage of heritability explained by the discovered loci in African Americans (from 10 % with ADP 2uM to 17 % with epinephrine). With a larger sample size, we probably would have discovered additional genetic variants with smaller effect sizes.

Assessment of ex vivo platelet function is labor intensive and very few cohorts have been generated with this phenotype; fewer still have substantial numbers of African Americans. In general, compared to the PGAP cohort, GeneSTAR participants had a higher incidence of CAD risk factors. Nevertheless, the minor allele frequencies (MAF) for each of the African American cohorts in this report were remarkably similar (Table 2). The GeneSTAR and PGAP studies also utilized the same platelet agonists and same genotyping platform, features of the study design that support the validity of our analyses. The replicated variants were not associated with fibrinogen levels in any cohort (Additional file 1: Table S1).

The rs12041331 SNP in *PEAR1* has previously been associated with epinephrine-and ADP-mediated platelet aggregation in European Americans and African Americans [13, 15]. *PEAR1* encodes a 1037 amino acid platelet cell surface receptor and, upon activation, an intracellular tyrosine residue is phosphorylated, followed by degranulation, amplification of the glycoprotein IIb/IIIa pathway and sustained platelet aggregation, most likely through the PI3K/Akt pathway [21, 22]. We now extend the association to include collagen-mediated platelet aggregation, findings confirmed in an independent group of African Americans. The variant identified by rs12041331 has been shown to regulate expression of PEAR1 protein in a dose-dependent fashion [15]. Taken together, these data suggest that PEAR1 levels are important effectors of platelet aggregation in both African and European Americans.

Of the five novel variants associated with platelet aggregation in the discovery sample, 2 were confirmed in at least one of our replication cohorts. Three novel variants did not replicate, suggesting they might be false positives. The most intriguing replicated variant was rs11202221 in *BMPR1A*; the G-allele of this SNP was associated with decreased ADP-induced platelet aggregation. This is of interest because BMPR1A has been implicated in vascular calcification as well as in the development of atherosclerosis [23], and platelets play a role in pathogenesis of the latter. *BMPR1A* encompasses 168 kb in the 10q23.2 region and encodes a 532 amino acid long single-pass cell surface receptor. This receptor belongs to the BMP receptor family of the transforming growth factor-beta (TGF-β) receptor superfamily and is expressed widely in various tissues. On ligand binding, BMPR1A activates intracellular signaling pathways, commonly leading to altered gene expression. Although BMPR1A has not been identified in platelets, several reports indicate it is expressed in megakaryocytes [24, 25], the bone marrow progenitor cell that produces platelets. Thus, the variants in *BMPR1A* that are associated with platelet aggregation could alter BMPR1A expression and/or function in megakaryocytes, which in turn could alter gene expression in signaling molecules mediating ADP-induced platelet aggregation. Alternatively, these *BMPR1A* SNPs could be in LD with other causative SNPs in either protein-coding or non-protein coding genes. Among the protein-coding genes near *BMPR1A*, transcripts of *MMRN2*, *GLUD1*, *WAPL*, and *PAPSS2* are present in platelets, however, only *GLUD1* (glutamate dehydrogenase 1) and *PAPSS2* (3'-phosphoadenosine 5'-phosphosulfate synthase 2) protein-products are present in platelets [26–28]. There are no microRNAs or lincRNAs within 500 kb of rs11202221.

It is intriguing that rs11202221 in *BMPR1A* did not replicate in European Americans. It is unlikely that this SNP is a false positive because 1) it replicated in PGAP African Americans, 2) the effect of the minor allele on platelet aggregation was the same direction in the two African American populations, and 3) there were 5 other SNPs in *BMPR1A* that showed association (p < 10^−5^) with ADP-induced platelet aggregation in both African American populations. LD patterns differ dramatically between European (CEU) and African (YRI) populations represented in the 1000 Genomes Project (see Additional file 1 Fig. S3). There is a 53 Kb block of LD including rs11202221 in the CEU reference population where variants are high in frequency (G allele frequency ~20 % at rs11202221, and MAF of most SNPs in the block are ~20 %). In contrast, no LD block in the YRI reference population includes rs11202221 and the allele frequency of the G allele is considerably lower (4 %). Given the tagging approach employed herein with the GWAS array, we speculate that rs11202221 tags the true 'causal' variant which itself may be low in frequency. Under this hypothesis, the 'causal' variant would consequently be tagged with higher correlation in the African American population in contrast to the European American population and therefore yield significant results in the African Americans but not European Americans. Future work will involve a targeted resequencing in the full dataset to fully examine all sequence-identified variants in this region and specifically test this hypothesis.

The second novel association with ADP-mediated aggregation is of an intergenic variant at 18q22.3 locus with ADP-mediated platelet aggregation. The 500 kb region on either side of this variant contains a few genes, but the transcript of only one, FBXO15 (which encodes F-box protein 15), has been reported at low levels in platelets. F-box proteins are important in substrate recognition by certain ubiquitin protein ligase complexes and thus are important in regulating protein degradation [29, 30]. While it is possible that this variant may affect protein degradation in platelets, the role of FBXO15 in platelet aggregation remains unexplored.

Different agonists and agonist concentrations were utilized to generate more refined platelet signaling pathway phenotypes. Collagen activates platelets via glycoprotein VI, which signals via an immunoreceptor tyrosine activation motif [31]. ADP induces platelet activation through the P2Y_12_ and P2Y_1_ G protein coupled receptors (GPCRs), while epinephrine activates platelets via a different GPCR, the α2A-adrenergic receptor [32]. Each of these GPCRs activates different G protein families, which in turn activate different sets of signaling molecules. Eventually, these different proximal signaling pathways converge to a final common pathway resulting in integrin αIIIbβ3 activation and platelet aggregation. Because SNPs in PEAR1 were associated with platelet aggregation induced by all three of these physiologic agonists, our data suggest a potential role for PEAR1 in a shared signaling pathway downstream of receptor-proximal signaling. Furthermore, ADP at low concentrations (e.g., 2 μM) induces rapid and reversible platelet aggregation through G_q_-coupled P2Y_1_, whereas high ADP concentrations (e.g., 10 μM) induce G_i_-coupled P2Y_12_ inhibition of adenyl cyclase and complete aggregation [33]. Thus, our findings that different loci were associated with different concentrations of ADP-induced platelet aggregation support hypotheses whereby *ALDH1L1-AS2, SUFU* and *BMPR1A* regulate primarily platelet G_q_ signaling and *SDK2* regulates primarily G_i_ signaling in platelets.

## Conclusions

In conclusion, we report here results of the first GWAS of agonist-induced platelet aggregation in African Americans. Our results confirm the importance of *PEAR1* in platelet biology and establish that variants in this gene affect platelet function in both African and European Americans. In addition, we have discovered and replicated novel loci associated with ADP-mediated platelet aggregation, one of which (rs11202221 in *BMPR1A*) may affect platelet function in African Americans but not in European Americans. Inhibition of ADP-induced platelet aggregation with thienopyridines is a mainstay of CAD treatment, and it will be important to consider race in the pharmacogenetics of these anti-platelet therapies. Further study of the loci discovered in this report may provide important insights into platelet biology and may identify targets for the development of novel anti-platelet agents.

## Methods

### Genetic Study of Atherosclerosis Risk (GeneSTAR) cohort

The design of GeneSTAR has been reported previously [10, 34]. Briefly, African American and European American probands with documented early-onset CAD were identified at the time of the event at ten Baltimore hospitals. Their apparently healthy family members (siblings, offspring of probands and siblings, and parents of the offspring) were enrolled. Eligible participants were interviewed by a nurse practitioner and self-reported their age and race. They underwent a cardiovascular history and physical examination and assessment of cardiovascular risk factors. Individuals with personal history of CAD, bleeding disorders, serious comorbidities, or who were taking anticoagulants, antiplatelet agents or nonsteriodal anti-inflammatory agents that could not be safely discontinued for two weeks prior to the study start were excluded. Individuals were also excluded if they had abnormal platelet count (<100,000/μL or > 500,000/μL) hematocrit (<30 %), or white blood cell count (>20,000/μL). The study was approved by the Johns Hopkins Institutional Review Board and all study participants gave informed consent (Additional file 1: Fig. S1).

### Platelet Genes and Physiology (PGAP) cohort

Healthy volunteers were recruited between 2001–2006 in Houston, Texas. The study protocol was approved by the Institutional Review Boards of Baylor College of Medicine and Thomas Jefferson University, and informed consent was obtained from all participants. Subjects were 18–80 years of age and were excluded if they had diabetes or hypertension, or had taken anti-platelet drugs within the past 10 days, anti-inflammatory drugs within the past 48 h, more than one prescribed medication (excluding oral contraceptives and hormone replacement therapy), or had been exposed to medication that affected the bone marrow. Eligible participants were interviewed by a study coordinator and self-reported their age and race. Hematocrit and platelet counts were in the normal range.

### Platelet aggregation studies

In both cohorts, blood was obtained by venipuncture and collected into EDTA (for complete blood cell counts) or 3.2 % sodium citrate (for platelet function testing). Platelet counts were determined by automated cell counter. Platelet-rich plasma was prepared from whole blood by centrifugation at 180 x *g* for 15 min, and platelet-poor plasma was prepared by centrifugation at 2000 x *g* for 10 min. Platelet counts were adjusted to 200,000/μL by diluting platelet-rich plasma with platelet-poor plasma.

Optical aggregometry with platelet-rich plasma was used to measure platelet aggregation (0-100 %) and maximal aggregation was determined within 5 min of stimulating samples with various platelet agonists. In GeneSTAR, platelets were stimulated with adenosine diphosphate (ADP 2 and 10 μM), arachidonic acid (1.6 mM), collagen (2 μg/mL) or epinephrine (2 μM). In PGAP, platelets were stimulated with ADP (4 μM), collagen (2 μg/mL), arachidonic acid (1.6 mM) and epinephrine (1.5 μM). As agonist doses were not identical for ADP and epinephrine in GeneSTAR and PGAP, we compared the platelet aggregation results with both doses of ADP from GeneSTAR with ADP 4 μM from PGAP and epinephrine 2 uM from GeneSTAR with epinephrine 1.5 μM from PGAP analyses.

### Genotype data and quality control

In GeneSTAR, genome-wide SNP genotyping was performed at deCODE Genetics, Inc. using the Human 1Mv1_C array from Illumina, Inc. with an average call rate per sample of 99.65 % (overall missing data rate = 0.35 %). Using 25 duplicate pairs, the reproducibility rate was >99.95 %. Samples that showed Mendelian errors > 5 % were excluded. We also excluded SNPs with call rate < 90 %, MAF <5 % and/or severe deviation from Hardy Weinberg equilibrium (p-value < 10^−6^) in the discovery sample.

In PGAP, genotyping was performed using the Illumina Human1M BeadChip at Baylor College of Medicine. Individuals with greater than 3 % missing genotypes, or average heterozygosity greater than 2 standard deviations from the mean were excluded. Any SNP locus with >5 % missing genotypes or deviation from Hardy Weinberg Equilibrium (p-value < 10^−6^) was removed. In the final analysis, only those SNPs were included in the analysis for which data were available in both the discovery and replication samples.

### Data analysis

Analyses were performed separately for the African Americans and European Americans in GeneSTAR. All variables were assessed for Gaussian distribution. Since the distribution response to epinephrine and collagen in both African American cohorts was bimodal, we dichotomized the phenotypes at the visual intersection of the two modal distributions. For all platelet aggregation phenotypes, we evaluated age- and sex-adjusted linear additive models. To account for intrafamilial relationships in GeneSTAR, linear mixed effects models were used to test the association under an additive model between a SNP and specific phenotype adjusted for age, sex and 2 principal components [13]. In our previous work with platelet aggregation phenotypes, we have found that only the first two principal components are associated with platelet phenotypes and that including more than two principal components in GWAS analyses does not provide additional information. The formulation of the mixed model follows in a matrix form: Y = XB + ZU + ε, where Y is an m × 1 vector of responses; X is an m × p design matrix of the fixed effects; B is the parameter p × 1 vector of fixed effects; Z is an m × q incidence matrix of random effects, and U is a q × 1 vector of random effects with E(U) = 0, and covariance matrix G; 0 is an m × 1 vector of random effects with E(0) = 0 and covariance matrix R. We tested whether the SNPs additive effects are different from zero, and especially we identified the highest significances. These models were run using PROC MIXED in SAS (v. 9.1.3 for Linux OS) with the option for EMPIRICAL variance and including the family identification number in the random effects to account for relatedness [35, 36] (SAS Institute, Cary, North Carolina, 1996). For collagen 2 μg/mL phenotypes, logistic models were used using generalized estimating equations for GeneSTAR. The data from PGAP was analyzed under additive models using R library GenABEL [37]. We adjusted for cryptic population structure using the method proposed by Chen and Abecasis as implemented in the “mmscore’ function in the GenABEL package [38]. The mmscore function uses the formula {((G - E[G]) V^−1^ × Y)^2^}/{(G - E[G]) V^−1^ (G - E[G])} where G is the vector of genotypes (coded 0, 1, 2) and E[G] is a vector of (strata-specific) mean genotypic values; V^−1^ is the inverse of the variance-covariance matrix at the maximum of polygenic model and Y are residuals after both the effect of covariates and the estimated polygenic effect (breeding values) are factored out [37]. We also used linear or logistic regression adjusting for age, sex, and 2 principal components and found similar results; hence we are presenting results obtained using mmscore function. In all three cohorts, we tested whether the SNP additive effects differed from zero. GWAS significance threshold was set at p-value < 5 × 10^−8^ and for replication, a Bonferroni adjusted p-value of < 0.008 was considered significant. Suggestive association between a variant and a phenotype was said to be present if association p-value was < 5 × 10^−6^. The African American cohort of the GeneSTAR study served as the discovery sample and the PGAP cohort served as a replication sample. As the PGAP sample size was small and hence had limited power, we also examined the European American cohort of the GeneSTAR as an additional replication sample. Due to the differences in the linkage disequilibrium pattern between populations of African and European descent, we also examined the association of the phenotype with nearby SNPs (within 250 kbp on either side) in the European American replication sample if the lead SNP in the African American discovery sample was not statistically significant in the African American or European American replication cohorts.

### Availability of supporting data

Based on participant consent forms, only aggregate data are available on dbGAP. Individual level data will be available using a Limited Access Agreement, which requires submission of an application and a study description to the GeneSTAR Study Steering Committee at The Johns Hopkins University. All data and all information will be fully deidentified according to our consent process. For further information, please visit: http://www.genestarstudy.com/For-Researchers.html.

## Additional file

Additional file 1:
**The supplemental material contains the following:** **Table S1.** Hemostatic characteristics across genotypes of replicated SNPs. **Table S2.** Loci with suggestive association findings in the GWAS of epinephrine-mediated platelet aggregation in African Americans. **Table S3.** Loci with suggestive association findings in the GWAS of collagen-mediated platelet aggregation in African Americans. **Table S4.** ADP-mediated Platelet Aggregation across Genotypes of the Two Novel Genetic Variants. **Table S5.** Loci with suggestive association findings in the GWAS of ADP 2 μM-mediated platelet aggregation in African Americans. **Table S6.** Loci with suggestive association findings in the GWAS of ADP 10 μM-mediated platelet aggregation in African Americans. **Table S7.** Loci with suggestive association findings in the GWAS of arachidonic acid-mediated platelet aggregation in African Americans. **Figure S1.** Study Design of the Genetic Study of Aspirin Responsiveness (GeneSTAR) and Platelet Genetics and Physiology (PGAP). **Figure S2.** Quantile-Quantile (QQ) plots with genomic inflation factors (λ)**. Figure S3.** Linkage disequilibrium plots of European descent population (CEU) and African descent population (YRI) based on the data from 1000 Genomes Project.

## Abbreviations

GWAS: Genome-wide association study
PGAP: Platelet genes and physiology
EDTA: Ethylenediaminetetraacetic acid
GeneSTAR: Genetic Study of Atherosclerosis Risk
SNP: single nucleotide polymorphisms
PEAR1: Platelet endothelial aggregation receptor 1
ADP: Adenosine diphosphate
ALDH1L1-AS2: Aldehyde dehydrogenase 1 family, member L1-antisense RNA 2
SUFU: Suppressor of fused homologue
BMPR1A: Bone morphogenetic protein receptor, type IA
SDK2: Sidekick cell adhesion molecule 2
FRMPD1: FERM and PDZ domain containing 1
CAD: coronary artery disease
MAF: Minor allele frequency
PI3K/Akt: phosphatidylinositol 3-kinase/protein kinase B
BMP: Bone morphogenetic protein
TGF-β: Transforming growth factor-beta
MMRN2: Multimerin 2
GLUD1: glutamate dehydrogenase 1
WAPL: Wings apart-like homolog
PAPSS2: 3'-phosphoadenosine 5'-phosphosulfate synthase 2
lincRNA: Long intervening noncoding RNA
SLC41A3: Solute carrier family 41, member 3
FBXO15: F-box protein 15

## References

[1] Nieswandt B, Pleines I, Bender M (2011). Platelet adhesion and activation mechanisms in arterial thrombosis and ischaemic stroke. J Thromb Haemost.

[2] Lievens D, von Hundelshausen P (2011). Platelets in atherosclerosis. Thromb Haemost.

[3] Yee DL, Sun CW, Bergeron AL, Dong JF, Bray PF (2005). Aggregometry detects platelet hyperreactivity in healthy individuals. Blood.

[4] Marcucci R, Gori AM, Paniccia R, Giusti B, Valente S, Giglioli C (2010). High on-treatment platelet reactivity by more than one agonist predicts 12-month follow-up cardiovascular death and non-fatal myocardial infarction in acute coronary syndrome patients receiving coronary stenting. Thromb Haemost.

[5] Breet NJ, van Werkum JW, Bouman HJ, Kelder JC, Ruven HJ, Bal ET (2010). Comparison of platelet function tests in predicting clinical outcome in patients undergoing coronary stent implantation. JAMA.

[6] Bordeaux BC, Qayyum R, Yanek LR, Vaidya D, Becker LC, Faraday N (2010). Effect of obesity on platelet reactivity and response to low-dose aspirin. Prev Cardiol.

[7] Faraday N, Yanek LR, Vaidya D, Kral B, Qayyum R, Herrera-Galeano JE (2009). Leukocyte count is associated with increased platelet reactivity and diminished response to aspirin in healthy individuals with a family history of coronary artery disease. Thromb Res.

[8] Vaidya D, Yanek LR, Faraday N, Moy TF, Becker LC, Becker DM (2009). Native platelet aggregation and response to aspirin in persons with the metabolic syndrome and its components. Metab Syndr Relat Disord.

[9] Bordeaux B, Yanek LR, Moy TF, White LW, Becker LC, Faraday N (2007). Casual chocolate consumption and inhibition of platelet function. Prev Cardiol.

[10] Becker DM, Segal J, Vaidya D, Yanek LR, Herrera-Galeano JE, Bray PF (2006). Sex differences in platelet reactivity and response to low-dose aspirin therapy. JAMA.

[11] Bray PF, Mathias RA, Faraday N, Yanek LR, Fallin MD, Herrera-Galeano JE (2007). Heritability of platelet function in families with premature coronary artery disease. J Thromb Haemost.

[12] Kunicki TJ, Nugent DJ (2010). The genetics of normal platelet reactivity. Blood.

[13] Johnson AD, Yanek LR, Chen MH, Faraday N, Larson MG, Tofler G (2010). Genome-wide meta-analyses identifies seven loci associated with platelet aggregation in response to agonists. Nat Genet.

[14] Casto AM, Feldman MW (2011). Genome-wide association study SNPs in the human genome diversity project populations: does selection affect unlinked SNPs with shared trait associations?. PLoS Genet.

[15] Faraday N, Yanek LR, Yang XP, Mathias R, Herrera-Galeano JE, Suktitipat B (2011). Identification of a specific intronic PEAR1 gene variant associated with greater platelet aggregability and protein expression. Blood.

[16] Thomas KL, Honeycutt E, Shaw LK, Peterson ED (2010). Racial differences in long-term survival among patients with coronary artery disease. Am Heart J.

[17] Berry JD, Dyer A, Cai X, Garside DB, Ning H, Thomas A (2012). Lifetime risks of cardiovascular disease. N Engl J Med.

[18] Marenberg ME, Risch N, Berkman LF, Floderus B, de Faire U (1994). Genetic susceptibility to death from coronary heart disease in a study of twins. N Engl J Med.

[19] Rissanen AM (1979). Familial occurrence of coronary heart disease: effect of age at diagnosis. Am J Cardiol.

[20] Rissanen AM (1979). Familial aggregation of coronary heart disease in a high incidence area (North Karelia, Finland). Br Heart J.

[21] Nanda N, Bao M, Lin H, Clauser K, Komuves L, Quertermous T (2005). Platelet endothelial aggregation receptor 1 (PEAR1), a novel epidermal growth factor repeat-containing transmembrane receptor, participates in platelet contact-induced activation. J Biol Chem.

[22] Kauskot A, Di Michele M, Loyen S, Freson K, Verhamme P, Hoylaerts MF (2012). A novel mechanism of sustained platelet alphaIIbbeta3 activation via PEAR1. Blood.

[23] Derwall M, Malhotra R, Lai CS, Beppu Y, Aikawa E, Seehra JS (2012). Inhibition of bone morphogenetic protein signaling reduces vascular calcification and atherosclerosis. Arterioscler Thromb Vasc Biol.

[24] Garimella R, Kacena MA, Tague SE, Wang J, Horowitz MC, Anderson HC (2007). Expression of bone morphogenetic proteins and their receptors in the bone marrow megakaryocytes of GATA-1(low) mice: a possible role in osteosclerosis. J Histochem Cytochem.

[25] Watkins NA, Gusnanto A, de Bono B, De S, Miranda-Saavedra D, Hardie DL (2009). A HaemAtlas: characterizing gene expression in differentiated human blood cells. Blood.

[26] Burkhart JM, Vaudel M, Gambaryan S, Radau S, Walter U, Martens L (2012). The first comprehensive and quantitative analysis of human platelet protein composition allows the comparative analysis of structural and functional pathways. Blood.

[27] Rowley JW, Oler AJ, Tolley ND, Hunter BN, Low EN, Nix DA (2011). Genome-wide RNA-seq analysis of human and mouse platelet transcriptomes. Blood.

[28] Bugert P, Kluter H (2006). Profiling of gene transcripts in human platelets: an update of the platelet transcriptome. Platelets.

[29] Kato M, Kito K, Ota K, Ito T (2010). Remodeling of the SCF complex-mediated ubiquitination system by compositional alteration of incorporated F-box proteins. Proteomics.

[30] Ho MS, Tsai PI, Chien CT (2006). F-box proteins: the key to protein degradation. J Biomed Sci.

[31] Watson SP, Gibbins J (1998). Collagen receptor signalling in platelets: extending the role of the ITAM. Immunol Today.

[32] Smyth SS, Woulfe DS, Weitz JI, Gachet C, Conley PB, Goodman SG (2009). G-protein-coupled receptors as signaling targets for antiplatelet therapy. Arterioscler Thromb Vasc Biol.

[33] Jin J, Kunapuli SP (1998). Coactivation of two different G protein-coupled receptors is essential for ADP-induced platelet aggregation. Proc Natl Acad Sci U S A.

[34] Qayyum R, Becker DM, Yanek LR, Moy TF, Becker LC, Faraday N (2008). Platelet inhibition by aspirin 81 and 325 mg/day in men versus women without clinically apparent cardiovascular disease. Am J Cardiol.

[35] Littell RC, Milliken GA, Stroup WW, Wolfinger RD. SAS® for Mixed Models. 2nd ed. Cary: SAS Institute; 2006.

[36] Suktitipat B, Mathias RA, Vaidya D, Yanek LR, Young JH, Becker LC (2012). The robustness of generalized estimating equations for association tests in extended family data. Hum Hered.

[37] Aulchenko YS, Ripke S, Isaacs A, van Duijn CM (2007). GenABEL: an R library for genome-wide association analysis. Bioinformatics.

[38] Chen WM, Abecasis GR (2007). Family-based association tests for genomewide association scans. Am J Hum Genet.

