# Supplementary material for: Genome-wide association study of platelet aggregation in African Americans
Source: BMC Genet. 2015 May 30;16:58. doi: 10.1186/s12863-015-0217-9 (PMC4448541; doi:10.1186/s12863-015-0217-9)
Supplement: Additional file 1: — The supplemental material contains the following: Table S1. Hemostatic characteristics across genotypes of replicated SNPs. Table S2. Loci with suggestive association findings in the GWAS of epinephrine-mediated platelet aggregation in African Americans. Table S3. Loci with suggestive association findings in the GWAS of collagen-mediated platelet aggregation in African Americans. Table S4. ADP-mediated Platelet Aggregation across Genotypes of the Two Novel Genetic Variants. Table S5. Loci with suggestive association findings in the GWAS of ADP 2 μM-mediated platelet aggregation in African Americans. Table S6. Loci with suggestive association findings in the GWAS of ADP 10 μM-mediated platelet aggregation in African Americans. Table S7. Loci with suggestive association findings in the GWAS of arachidonic acid-mediated platelet aggregation in African Americans. Figure S1. Study Design of the Genetic Study of Aspirin Responsiveness (GeneSTAR) and Platelet Genetics and Physiology (PGAP). Figure S2. Quantile-Quantile (QQ) plots with genomic inflation factors (λ). Figure S3. Linkage disequilibrium plots of European descent population (CEU) and African descent population (YRI) based on the data from 1000 Genomes Project. [file 12863_2015_217_MOESM1_ESM.pdf]

# SUPPLEMENTAL MATERIAL

The supplemental material contains the following:

1. **Supplementary Table 1:** Hemostatic characteristics across genotypes of replicated SNPs
2. **Supplementary Table 2:** Loci with suggestive association findings in the GWAS of epinephrine-mediated platelet aggregation in African Americans
3. **Supplementary Table 3:** Loci with suggestive association findings in the GWAS of collagen-mediated platelet aggregation in African Americans
4. **Supplementary Table 4:** ADP-mediated Platelet Aggregation across Genotypes of the Two Novel Genetic Variants
5. **Supplementary Table 5:** Loci with suggestive association findings in the GWAS of ADP 2µM-mediated platelet aggregation in African Americans
6. **Supplementary Table 6:** Loci with suggestive association findings in the GWAS of ADP 10µM-mediated platelet aggregation in African Americans
7. **Supplementary Table 7:** Loci with suggestive association findings in the GWAS of arachidonic acid-mediated platelet aggregation in African Americans
8. **Supplemental Figure 1:** Study Design of the Genetic Study of Aspirin Responsiveness (GeneSTAR) and Platelet Genetics and Physiology (PGAP)
9. **Supplementary Figure 2:** Quantile-Quantile (QQ) plots with genomic inflation factors (λ)
10. **Supplemental Figure 3:** Linkage disequilibrium plots of European descent population (CEU) and African descent population (YRI) based on the data from 1000 Genomes Project

**Supplementary Table 1:** Hemostatic characteristics across genotypes of replicated SNPs

| **rs12041331** | | | | |
|---|---|---|---|---|
| ***Genotypes*** | **AA (N=110)** | **GA (N=367)** | **GG (N=348)** | **P-value*** |
| Platelet Count ($10^9$/L) | 277.8 (72.8) | 274.2 (70.8) | 264.4 (66.0) | 0.07 |
| Mean Platelet Volume ($10^{-15}$/L) | 7.7 (0.84) | 8.0 (0.90) | 8.0 (0.87) | 0.06 |
| Fibrinogen (mg/dL) | 428.6 (142.1) | 422.4 (122.3) | 406.2 (127.8) | 0.34 |
| Collagen-induced Platelet Aggregation (%) | 44.2 (35.2) | 62.7 (30.9) | 69.2 (26.1) | $2.74 \times 10^{-11}$ |
| Epinephrine-induced Platelet Aggregation (%) | 27.3 (33.3) | 46.0 (35.0) | 62.0 (33.8) | $2.82 \times 10^{-12}$ |
| ADP(2µM)-induced Platelet Aggregation (%) | 30.7 (25.6) | 37.4 (27.8) | 47.1 (28.2) | $5.8 \times 10^{-9}$ |
| ADP(10µM)-induced Platelet Aggregation (%) | 69.1 (19.5) | 74.4 (18.7) | 80.4 (14.7) | $3.2 \times 10^{-10}$ |
| **rs11202221** | | | | |
| ***Genotypes*** | **GG (N=3)** | **TG (N=114)** | **TT (N=706)** | **P-value*** |
| Platelet Count ($10^9$/L) | 245.0 (72.5) | 274.3 (64.7) | 269.9 (69.9) | 0.86 |
| Mean Platelet Volume ($10^{-15}$/L) | 7.7 (0.83) | 7.8 (0.95) | 7.9 (0.88) | 0.41 |
| Fibrinogen (mg/dL) | 359.5 (157.7) | 414.7 (131.1) | 416.4 (127.1) | 0.30 |
| Collagen-induced Platelet Aggregation (%) | 55.3 (44.5) | 55.8 (32.2) | 64.2 (30.2) | 0.09 |
| Epinephrine-induced Platelet Aggregation (%) | 18.7 (6.5) | 41.8 (34.1) | 51.9 (36.4) | 0.12 |
| ADP(2µM)-induced Platelet Aggregation (%) | 28.7 (11.0) | 29.5 (23.1) | 42.6 (28.8) | $4.8 \times 10^{-8}$ |
| ADP(10µM)-induced Platelet Aggregation (%) | 67.3 (24.0) | 71.5 (21.7) | 77.0 (16.9) | 0.01 |
| **rs6566765** | | | | |
| ***Genotypes*** | **CC (N=297)** | **CT (N=403)** | **TT (N=125)** | **P-value*** |
| Platelet Count ($10^9$/L) | 265.9 (62.8) | 272.9 (70.1) | 274.0 (80.0) | 0.44 |
| Mean Platelet Volume ($10^{-15}$/L) | 8.0 (0.88) | 7.9 (0.86) | 7.9 (0.98) | 0.95 |
| Fibrinogen (mg/dL) | 414.1 (131.9) | 414.9 (124.5) | 426.4 (127.5) | 0.62 |
| Collagen-induced Platelet Aggregation (%) | 59.0 (33.1) | 65.1 (28.6) | 65.7 (30.1) | 0.05 |
| Epinephrine-induced Platelet Aggregation (%) | 45.8 (37.1) | 51.1 (35.8) | 57.9 (33.8) | 0.15 |
| ADP(2µM)-induced Platelet Aggregation (%) | 37.0 (28.2) | 41.8 (28.4) | 45.0 (27.3) | 0.03 |
| ADP(10µM)-induced Platelet Aggregation (%) | 72.8 (19.0) | 76.9 (17.5) | 82.0 (12.9) | $3.59 \times 10^{-8}$ |

***Abbreviations:*** SNP = single nucleotide polymorphism**;** ADP = adenosine diphosphate

*Data is represented as mean and standard deviation. P-values for collagen and epinephrine-induced platelet aggregation are from dichotomized phenotypes using logistic models, other P-values are from linear regression. All tests were adjusted for familial correlation.

**Supplementary Table 2:** Loci with suggestive association findings in the GWAS of epinephrine-mediated platelet aggregation in African Americans

| | | | GeneSTAR | | | PGAP | | | GeneSTAR (EA) | | |
|---|---|---|---|---|---|---|---|---|---|---|---|
| **SNP** | **Position** | **Gene** | **β(SE)** | **P-value** | **MAF** | **β(SE)** | **P-value** | **MAF** | **β(SE)** | **P-value** | **MAF** |
| rs10803355_T | 1:15327779 | C1orf126 | -0.63 (0.13) | $3.43 \times 10^{-6}$ | 26.0% | 0.11 (0.05) | 0.03 | 30.4% | -0.07 (0.12) | 0.26 | 29.9% |
| rs922987_G | 2:217119491 | | -0.52 (0.11) | $1.28 \times 10^{-6}$ | 41.7% | -0.07(0.05) | 0.19 | 33.3% | -0.09 (0.09) | 0.29 | 49.8% |
| rs7000642_C | 8:64591068 | | -0.53 (0.11) | $1.30 \times 10^{-6}$ | 40.6% | 0.001 (0.05) | 0.98 | 41.6% | -0.003 (0.10) | 0.49 | 65.9% |
| rs12313736_A | 12:26275226 | SSPN | 0.63 (0.13) | $2.77 \times 10^{-6}$ | 17.7% | -0.07 (0.06) | 0.29 | 22.7% | -0.001(0.23) | 0.50 | 4.9% |
| rs12708769_T | 16:9077749 | | -0.51 (0.11) | $3.60 \times 10^{-6}$ | 28.1% | 0.07 (0.06) | 0.20 | 37.4% | -0.06 (0.10) | 0.53 | 27.0% |
| rs9889955_G | 17:69072972 | SDK2 | -0.67 (0.13) | $7.54 \times 10^{-7}$ | 24.0% | 0.07 (0.06) | 0.25 | 25.8% | 0.11 (0.11) | 0.30 | 19.2% |

***Abbreviations:*** SNP = single nucleotide polymorphism; β= regression coefficient; SE = standard error; GeneSTAR = Genetic Study of Atherosclerosis; PGAP = Platelet Gene and Physiology; MAF = minor allele frequency; ADP = adenosine diphosphate

Note: In PGAP the epinephrine concentration of 1.5 µM was used.

**Supplementary Table 3:** Loci with suggestive association findings in the GWAS of collagen-mediated platelet aggregation in African Americans

| | | | GeneSTAR (AA) | | | PGAP | | | GeneSTAR (EA) | | |
|---|---|---|---|---|---|---|---|---|---|---|---|
| **SNP** | **Position** | **Gene** | **β(SE)** | **P-value** | **MAF** | **β(SE)** | **P-value** | **MAF** | **β(SE)** | **P-value** | **MAF** |
| rs1766285_T | 1:77160219 | ST6GALNAC5 | -0.58 (0.12) | $1.04 \times 10^{-6}$ | 28.5% | 0.004 (0.63) | 0.99 | 28.8% | 0.13 (0.10) | 0.17 | 31.2% |
| rs16992175_C | 4:36043106 | | -0.820(0.15) | $1.04 \times 10^{-7}$ | 10.9% | -1.01 (1.01) | 0.31 | 8.6% | -0.33 (0.40) | 0.28 | 7.3% |
| rs7728135_A | 5:67134354 | | -0.76 (0.15) | $8.98 \times 10^{-7}$ | 18.0% | 0.21 (0.71) | 0.76 | 17.7% | -0.03 (0.13) | 0.39 | 23.7% |
| rs6951019_T | 7:673749 | PRKAR1B | -0.87 (0.19) | $3.07 \times 10^{-6}$ | 10.4% | 2.24 (0.84) | $7.8 \times 10^{-3}$ | 13.1% | 1.08 (1.05) | 0.24 | 8.3% |
| rs4590481_C | 8:72877955 | | -0.67 (0.14) | $1.38 \times 10^{-6}$ | 45.0% | 0.21 (0.57) | 0.71 | 48.5% | -0.09 (0.14) | 0.32 | 21.4% |
| rs2387916_T | 15:91068340 | LOC388177 | -0.78 (0.16) | $1.68 \times 10^{-6}$ | 15.4% | -0.26 (0.87) | 0.76 | 14.1% | 0.04 (0.09) | 0.35 | 50.6% |

***Abbreviations:*** SNP = single nucleotide polymorphism; β= regression coefficient; SE = standard error; GeneSTAR = Genetic Study of Atherosclerosis; PGAP = Platelet Gene and Physiology; MAF = minor allele frequency; AA = African Americans; EA = European Americans

Note: In PGAP the collagen concentration of 2.5 µg/mL was used.

**Supplementary Table 4:** ADP-mediated Platelet Aggregation across Genotypes of the Two Novel Genetic Variants.

<table>
<tr><th colspan="2">Genotypes</th><th rowspan="2">N</th><th rowspan="2">Allele Dose*</th><th rowspan="2">ADP 2 uM (%)</th><th rowspan="2">ADP 10 uM (%)</th></tr>
<tr><td>rs6566765</td><td>rs11202221</td></tr>
<tr><td>T/T</td><td>T/T</td><td>109</td><td>0</td><td>46.6</td><td>81.6</td></tr>
<tr><td>T/T</td><td>T/G</td><td>15</td><td rowspan="2">1</td><td rowspan="2">43.5</td><td rowspan="2">78.2</td></tr>
<tr><td>C/T</td><td>T/T</td><td>347</td></tr>
<tr><td>T/T</td><td>G/G</td><td>1</td><td rowspan="3">2</td><td rowspan="3">37.0</td><td rowspan="3">73.0</td></tr>
<tr><td>C/T</td><td>T/G</td><td>54</td></tr>
<tr><td>C/C</td><td>T/T</td><td>250</td></tr>
<tr><td>C/T</td><td>G/G</td><td>1</td><td rowspan="2">3</td><td rowspan="2">27.9</td><td rowspan="2">69.0</td></tr>
<tr><td>C/C</td><td>T/G</td><td>45</td></tr>
<tr><td>C/C</td><td>G/G</td><td>1</td><td>4</td><td>28</td><td>41</td></tr>
<tr><td colspan="4">P-value for trend</td><td>$4.55 \times 10^{-6}$</td><td>$4.58 \times 10^{-9}$</td></tr>
</table>

* Allele dose was calculated by the number of alleles associated with decreased platelet aggregation; C-allele for rs6566765 and G-allele for rs11202221.

**Supplementary Table 5:** Loci with suggestive association findings in the GWAS of ADP 2μM-mediated platelet aggregation in African Americans

| | | | GeneSTAR (AA) | | | PGAP | | | GeneSTAR (EA) | | |
|---|---|---|---|---|---|---|---|---|---|---|---|
| **SNP** | **Position** | **Gene** | **β(SE)** | **P-value** | **MAF** | **β(SE)** | **P-value** | **MAF** | **β(SE)** | **P-value** | **MAF** |
| rs2760480_T | 1:58975709 | | -7.10 (1.36) | $2.73 \times 10^{-7}$ | 44.8% | 3.70 (2.67) | 0.10 | 49.6% | -1.61 (1.32) | 0.23 | 41.8% |
| rs11487952_C | 1:74968400 | CRYZ | 6.39 (1.37) | $4.22 \times 10^{-6}$ | 47.7% | -2.40 (2.30) | 0.30 | 37.3% | - | - | - |
| rs12066611_C | 1:96548296 | | 9.73 (1.98) | $1.13 \times 10^{-6}$ | 9.4% | 3.01 (3.33) | 0.37 | 15.0% | - | - | - |
| rs1446877_A | 2:23069767 | | -8.01 (1.53) | $2.49 \times 10^{-7}$ | 42.7% | -1.27 (2.21) | 0.56 | 36.4% | -1.06 (1.40) | 0.45 | 80.3% |
| rs4252027_C | 2:113607229 | IL1RN | -11.67 (2.40) | $1.57 \times 10^{-6}$ | 6.4% | 0.04 (4.89) | 0.99 | 6.4% | | | |
| rs4683235_T | 3:46476935 | LTF | -9.35 (1.74) | $1.30 \times 10^{-7}$ | 19.8% | -2.73 (3.11) | 0.38 | 14.8% | -3.30 (2.09) | 0.11 | 8.3% |
| rs13072722 | 3:115907655 | ZBTB20 | 6.72 (1.41) | $2.54 \times 10^{-6}$ | 39.1% | -3.36 (2.56) | 0.20 | 35.5% | | | |
| rs6847949_A | 4:153368734 | | -7.47 (1.52) | $1.20 \times 10^{-6}$ | 26.0% | -1.60 (2.68) | 0.55 | 18.2% | -1.56 (1.47) | 0.29 | 22.6% |
| rs3846872_G | 6:37172645 | | -7.85 (1.66) | $3.06 \times 10^{-6}$ | 18.8% | 3.63 (2.62) | 0.16 | 26.4% | - | - | - |
| rs16875446_C | 8:108062827 | | -9.52 (2.04) | $3.93 \times 10^{-6}$ | 11.5% | -0.03 (3.24) | 0.99 | 13.2% | - | - | - |
| rs12220754_A | 10:2870121 | | 11.80 (2.32) | $5.46 \times 10^{-7}$ | 5.2% | 2.32 (4.58) | 0.61 | 6.8% | -0.94 (1.81) | 0.60 | 13.3% |
| rs4934272_T | 10:88567074 | BMPR1A | -11.06 (2.15) | $3.96 \text{ X } 10^{-7}$ | 10.4% | -16.39 (3.8) | $1.46 \times 10^{-5}$ | 9.7% | - | - | - |
| rs7095025_T | 10:88569340 | BMPR1A | -11.10 (2.16) | $3.66 \text{ X } 10^{-7}$ | 10.4% | -16.39 (3.8) | $1.46 \times 10^{-5}$ | 9.7% | -0.02 (1.38) | 0.99 | 75.3% |
| rs4934275_C | 10:88607372 | BMPR1A | -12.85 (2.39) | $1.19 \text{ X } 10^{-7}$ | 9.4% | -16.01 (3.6) | $7.52 \times 10^{-5}$ | 9.1% | 0.08 (1.75) | 0.96 | 85.4% |

| | | | | | | | | | | | |
|---|---|---|---|---|---|---|---|---|---|---|---|
| rs11202232_G | 10:88612333 | BMPR1A | -12.85 (2.39) | 1.19 X $10^{-7}$ | 9.4% | -17.75 (4.1) | 1.26 x $10^{-5}$ | 8.1% | 0.08 (1.75) | 0.96 | 85.4% |
| rs10887666_T | 10:88649027 | BMPR1A | -12.85 (2.39) | 1.19 X $10^{-7}$ | 9.4% | -16.92 (4.3) | 9.13 x $10^{-5}$ | 7.2% | 0.08 (1.75) | 0.96 | 85.4% |
| rs2686332_A | 12:24283633 | SOX5 | -9.77 (2.11) | 4.54 x $10^{-6}$ | 11.5% | 1.85 (4.37) | 0.67 | 6.8% | -0.36 (1.29) | 0.78 | 28.5% |
| rs10492301_C | 12:67180245 | | 10.36 (2.02) | 4.24 x $10^{-7}$ | 9.4% | -3.82 (3.31) | 0.25 | 13.1% | 1.45 (1.12) | 0.20 | 34.5% |
| rs28408948_G | 15:79785446 | | 10.39 (2.23) | 4.21 x $10^{-6}$ | 17.0% | 0.47 (3.31) | 0.89 | 18.1% | - | - | - |
| rs4782624_G | 16:83022667 | ATP2C2 | 7.805 (1.66) | 3.31 x $10^{-6}$ | 24.0% | -3.33 (2.34) | 0.15 | 31.8% | 0.63 (2.78) | 0.82 | 7.5% |
| rs12604055_T | 17:29122399 | ACCN1 | -12.16 (2.53) | 2.01 x $10^{-6}$ | 5.2% | 3.95 (4.26) | 0.35 | 7.7% | -1.62 (2.10) | 0.44 | 7.9% |
| rs9912932_A | 17:78220928 | RAB40B | -8.62 (1.84) | 3.48 x $10^{-6}$ | 12.5% | 1.16 (2.96) | 0.69 | 18.2% | -1.23 (1.23) | 0.31 | 32.1% |

***Abbreviations:*** SNP = single nucleotide polymorphism; β= regression coefficient; SE = standard error; GeneSTAR = Genetic Study of Atherosclerosis; PGAP = Platelet Gene and Physiology; MAF = minor allele frequency; ADP = adenosine diphosphate; AA = African Americans; EA = European Americans

Note: In PGAP the ADP concentration of 4 uM was used.

**Supplementary Table 6:** Loci with suggestive association findings in the GWAS of ADP 10μM-mediated platelet aggregation in African Americans

| | | | GeneSTAR (AA) | | | PGAP | | | GeneSTAR (EA) | | |
|---|---|---|---|---|---|---|---|---|---|---|---|
| **SNP** | **Position** | **Gene** | **β(SE)** | **P-value** | **MAF** | **β(SE)** | **P-value** | **MAF** | **β(SE)** | **P-value** | **MAF** |
| rs17039923_C | 2:50211959 | NRXN1 | 4.79 (0.96) | $9.43 \times 10^{-7}$ | 24.0% | -2.70 (2.53) | 0.29 | 25.8% | | | |
| rs7638187_T | 3:3152835 | TRNT1 | 6.19 (1.30) | $2.53 \times 10^{-6}$ | 8.3% | 1.53 (3.72) | 0.68 | 8.9% | 0.95 (0.79) | 0.15 | 16.8% |
| rs1076425_C | 3:52800502 | ITIH1 | 4.96 (1.00) | $1.01 \times 10^{-6}$ | 28.1% | -3.96 (2.96) | 0.18 | 22.9% | 0.62 (0.65) | 0.28 | 36.5% |
| rs6778453_T | 3:113653472 | | 6.46 (1.38) | $3.60 \times 10^{-6}$ | 5.2% | -7.35 (4.72) | 0.12 | 6.4% | -1.43 (1.47) | 0.33 | 7.4% |
| rs3792293_G | 3:168935198 | PDCD10 | -4.45 (0.91) | $1.34 \times 10^{-6}$ | 48.9% | 0.06 (2.19) | 0.98 | 41.9% | 0.16 (0.62) | 0.79 | 26.6% |
| rs1895742_C | 4:110206737 | COL25A1 | 4.58 (0.91) | $4.29 \times 10^{-6}$ | 13.5% | -6.73 (3.19) | 0.04 | 12.7% | -0.11 (0.73) | 0.87 | 28.7% |
| rs6877160_G | 5:178157153 | | 4.50 (0.96) | $3.73 \times 10^{-6}$ | 18.8% | 3.91 (2.47) | 0.11 | 24.2% | -0.14 (0.68) | 0.84 | 30.9% |
| rs17636617_A | 6:37609640 | | 6.53 (1.35) | $1.88 \times 10^{-6}$ | 5.2% | -2.47 (4.51) | 0.58 | 5.9% | 1.15 (1.16) | 0.32 | 8.3% |
| rs2076473_C | 6:56114802 | COL21A1 | 4.42 (0.93) | $2.54 \times 10^{-6}$ | 25.0% | -2.29 (2.51) | 0.36 | 27.1% | 0.57 (0.59) | 0.33 | 57.5% |
| rs2791829_C | 6:102399942 | GRIK2 | -5.93 (1.15) | $3.72 \times 10^{-7}$ | 13.5% | -1.66 (3.55) | 0.64 | 11.9% | - | - | - |
| rs2049924_T | 6:113858994 | | 4.52 (0.89) | $4.94 \times 10^{-7}$ | 26.0% | -0.45 (2.59) | 0.86 | 21.2% | -1.32 (0.72) | 0.07 | 37.5% |
| rs750693_C | 9:37723925 | FRMPD1 | 5.32 (1.12) | $2.76 \times 10^{-6}$ | 10.4% | -1.68 (3.75) | 0.65 | 10.2% | 1.75 (0.71) | 0.01 | 29.1% |
| rs1591958_A | 10:82052641 | | 4.26 (0.86) | $1.15 \times 10^{-6}$ | 38.5% | 2.21 (2.48) | 0.37 | 33.2% | 0.91 (0.71) | 0.20 | 18.9% |
| rs16928081_T | 11:2277542 | | 5.32 (1.13) | $3.44 \times 10^{-6}$ | 13.5% | 4.00 (3.89) | 0.30 | 8.9% | | | |
| rs1506525_G | 11:32001522 | | 4.45 (0.92) | $1.61 \times 10^{-6}$ | 24.0% | -1.46 (2.34) | 0.53 | 34.3% | -0.21 (1.23) | 0.87 | 17.5% |

| | | | | | | | | | | | |
|---|---|---|---|---|---|---|---|---|---|---|---|
| rs17755728_C | 11:78691521 | | 4.51 (0.97) | $4.38 \times 10^{-6}$ | 11.5% | 3.07 (2.86) | 0.28 | 19.1% | 0.52 (0.85) | 0.54 | 15.9% |
| rs6491870_C | 13:103993236 | | 4.77 (0.99) | $2.03 \times 10^{-6}$ | 36.5% | 2.23 (2.20) | 0.31 | 41.9% | 0.62 (0.69) | 0.37 | 28.8% |
| rs160475_G | 14:58205393 | | 4.83 (1.02) | $2.95 \times 10^{-6}$ | 24.0% | 2.17 (2.56) | 0.40 | 31.8% | -1.00 (0.97) | 0.37 | 10.9% |
| rs371125_G | 15:56460289 | | 6.78 (1.36) | $9.28 \times 10^{-7}$ | 10.4% | 1.33 (4.37) | 0.76 | 7.6% | | | |
| rs244401_A | 17:50675059 | | 6.75 (1.45) | $3.89 \times 10^{-6}$ | 7.3% | 5.08 (4.89) | 0.30 | 5.9% | -0.48 (0.72) | 0.51 | 19.2% |
| rs12159423_G | 22:20903637 | IGL locus | 4.71 (0.96) | $1.23 \times 10^{-6}$ | 24.0% | 4.25 (2.95) | 0.15 | 18.2% | 0.60 (1.11) | 0.58 | 7.5% |

***Abbreviations:*** SNP = single nucleotide polymorphism; β= regression coefficient; SE = standard error; GeneSTAR = Genetic Study of Atherosclerosis; PGAP = Platelet Gene and Physiology; MAF = minor allele frequency; ADP = adenosine diphosphate; AA = African Americans; EA = European Americans

Note: In PGAP an ADP concentration of 4 uM was used.

**Supplementary Table 7:** Loci with suggestive association findings in the GWAS of arachidonic acid-mediated platelet aggregation in African Americans

| | | | GeneSTAR (AA) | | | PGAP | | | GeneSTAR (EA) | | |
|---|---|---|---|---|---|---|---|---|---|---|---|
| **SNP** | **Position** | **Gene** | **β(SE)** | **P-value** | **MAF** | **β(SE)** | **P-value** | **MAF** | **β(SE)** | **P-value** | **MAF** |
| rs10921542_C | 1:192591090 | | -3.10 (0.69) | $1.12 \times 10^{-6}$ | 9.4% | 8.17 (8.21) | 0.32 | 6.5% | -0.52 (0.80) | 0.51 | 9.0% |
| rs10204598_A | 2:38205473 | Near CYP1B1-AS1 | 3.08 (0.61) | $7.65 \times 10^{-7}$ | 15.5% | -2.04 (5.34) | 0.70 | 18.7% | -1.09 (0.59) | 0.06 | 12.2% |
| rs11745148_T | 5:12234811 | | -3.11 (0.63) | $1.39 \times 10^{-6}$ | 17.2% | 6.72 (4.98) | 0.18 | 19.6% | 0.72 (0.41) | 0.08 | 34.3% |
| rs6554742_A | 5:12714704 | | 2.82 (0.54) | $3.06 \times 10^{-7}$ | 27.7% | 5.33 (4.27) | 0.21 | 29.9% | -0.11 (0.52) | 0.82 | 14.3% |
| rs2055437_T | 5:81998598 | | -2.39 (0.49) | $1.91 \times 10^{-6}$ | 31.1% | 5. 10 (4.61) | 0.28 | 30.8% | 0.65 (0.68) | 0.34 | 22.8% |
| rs4720990_G | 7:11488680 | THSD7A | -2.37 (0.51) | $4.71 \times 10^{-6}$ | 31.2% | -0.75 (4.21) | 0.86 | 34.1% | -0.02 (0.39) | 0.96 | 35.9% |
| rs10949695_C | 7:157553706 | PTPRN2 | 3.09 (0.66) | $3.69 \times 10^{-6}$ | 15.4% | 6.92 (5.39) | 0.20 | 18.6% | -0.67 (0.44) | 0.13 | 25.7% |
| rs7322119_A | 13:85383398 | | 2.48 (0.52) | $2.85 \times 10^{-6}$ | 25.2% | 1.62 (4.28) | 0.70 | 29.2% | 0.93 (0.61) | 0.13 | 14.8% |
| rs948799_T | 18:70004052 | Near TIMM21 | 2.87 (0.60) | $2.89 \times 10^{-6}$ | 19.3% | -1.79 (4.93) | 0.72 | 17.8% | - | - | - |
| rs3787563_T | 20:352916 | RBCK1 | -2.36 (0.51) | $4.91 \times 10^{-6}$ | 52.7% | 4.01 (4.03) | 0.31 | 48.1% | 0.08 (0.42) | 0.84 | 32.6% |

***Abbreviations:*** SNP = single nucleotide polymorphism; β= regression coefficient; SE = standard error; GeneSTAR = Genetic Study of Atherosclerosis; PGAP = Platelet Gene and Physiology; MAF = minor allele frequency; AA = African Americans; EA = European Americans

Note: In PGAP the arachidonic acid concentration of 0.5 mM was used.

**Supplemental Figure 1:** Study Design of the Genetic Study of Aspirin Responsiveness (GeneSTAR) and Platelet Genetics and Physiology (PGAP)

***Abbreviations:*** GeneSTAR-AA= African American cohort of the GeneSTAR study; GeneSTAR-EA = European American cohort of the GeneSTAR study; CAD = coronary artery disease; WBC = white blood count; GWAS = genome-wide association study

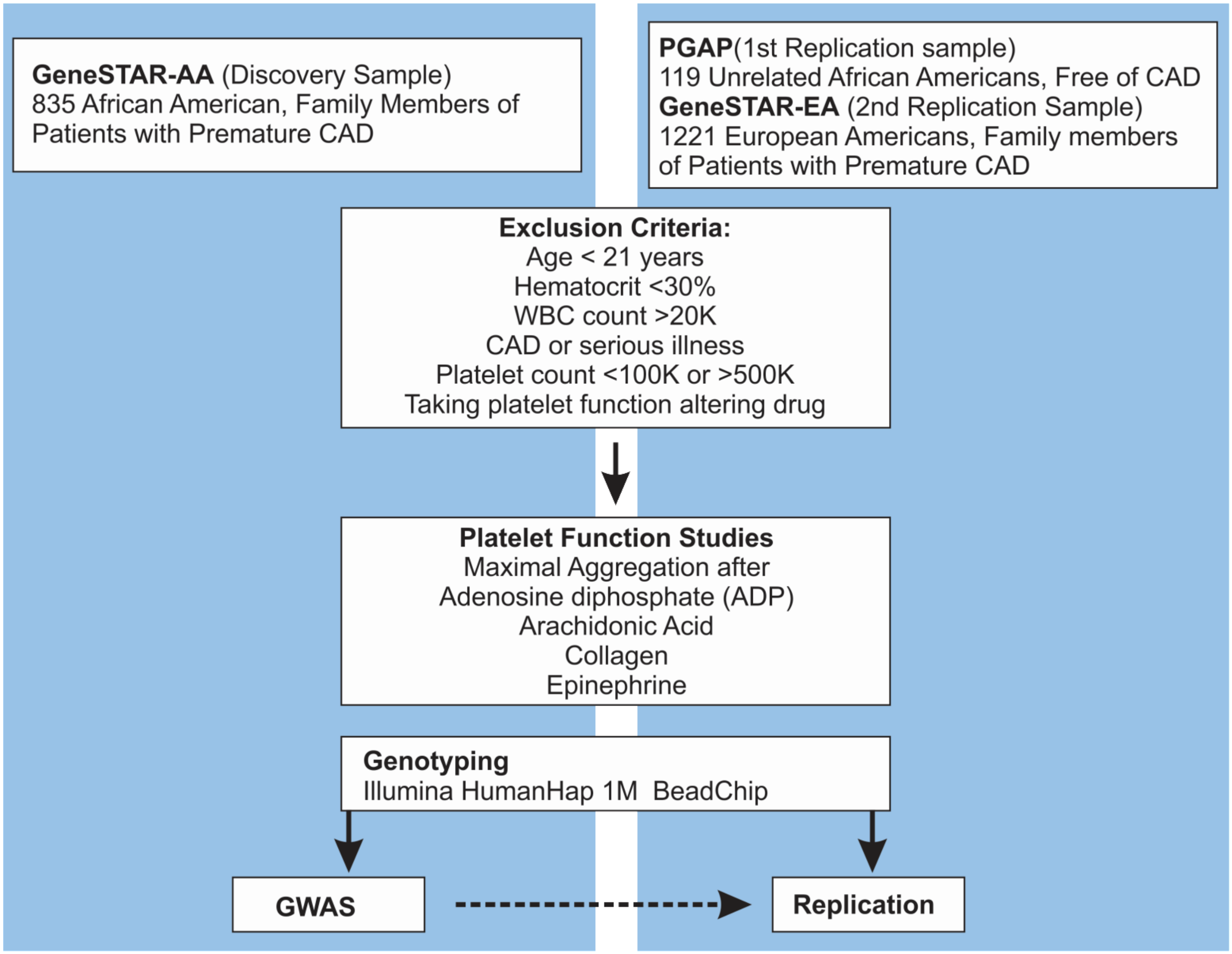

**Supplementary Figure 2:** Quantile-Quantile (QQ) plots with genomic inflation factors (λ)

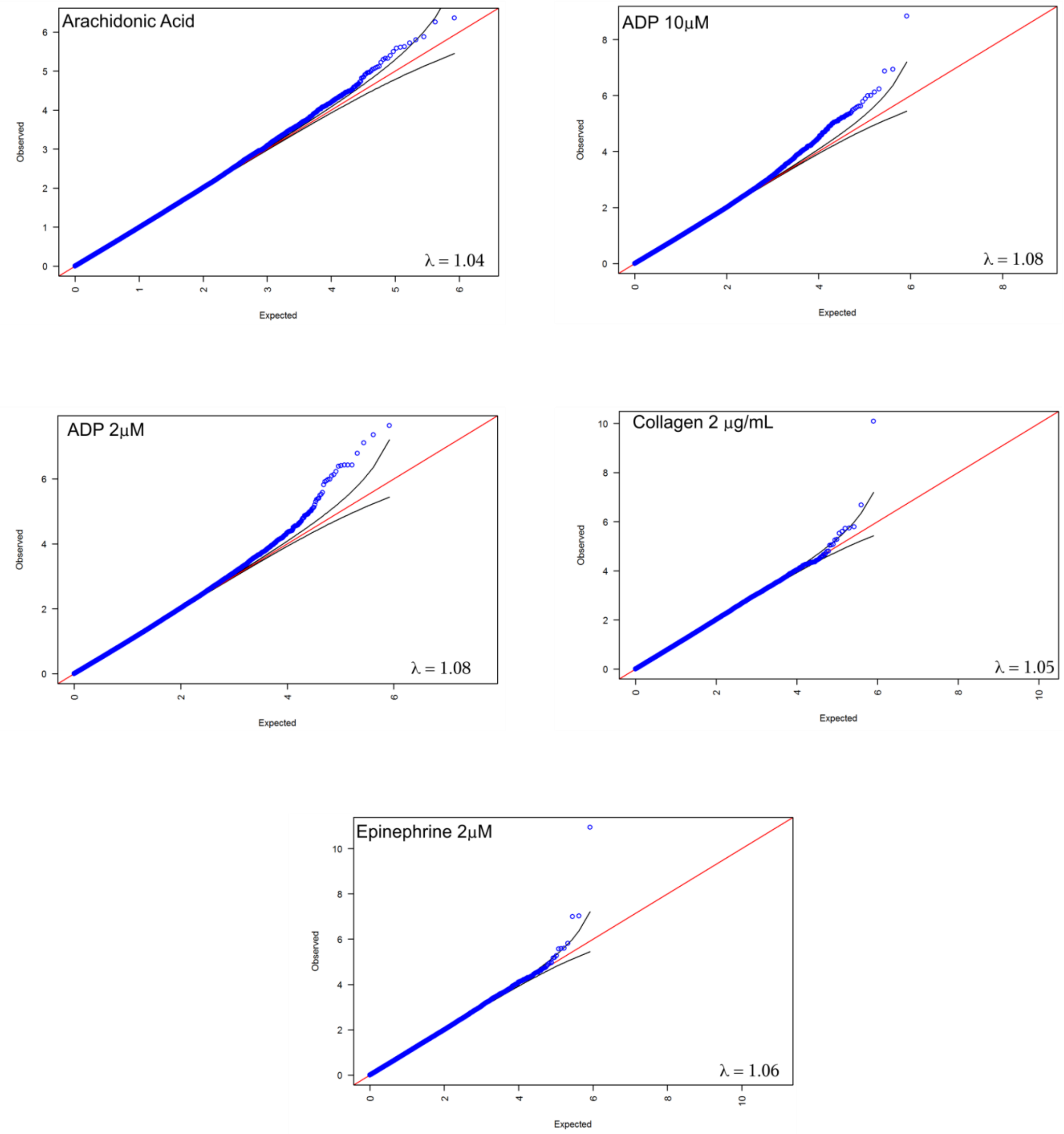

**Supplemental Figure 3:** Linkage disequilibrium plots of European descent population (CEU) and African descent population (YRI) based on the data from 1000 Genomes Project

**Top Panel:** LD structure in a 200kb region centered on rs1120221 in the low coverage sequence data from the Thousand Genomes Project reveals extensive LD in the CEU compared to the YRI populations. **Bottom panel:** Zoomed in region of a 53 kb LD block that includes rs1120221 (yellow arrow) with an MAF of 20% in the CEU data. In contrast the MAF at rs1120221 in the YRI is only 4% and as shown by the yellow arrow, it does not fall into any LD blocks in the YRI data.

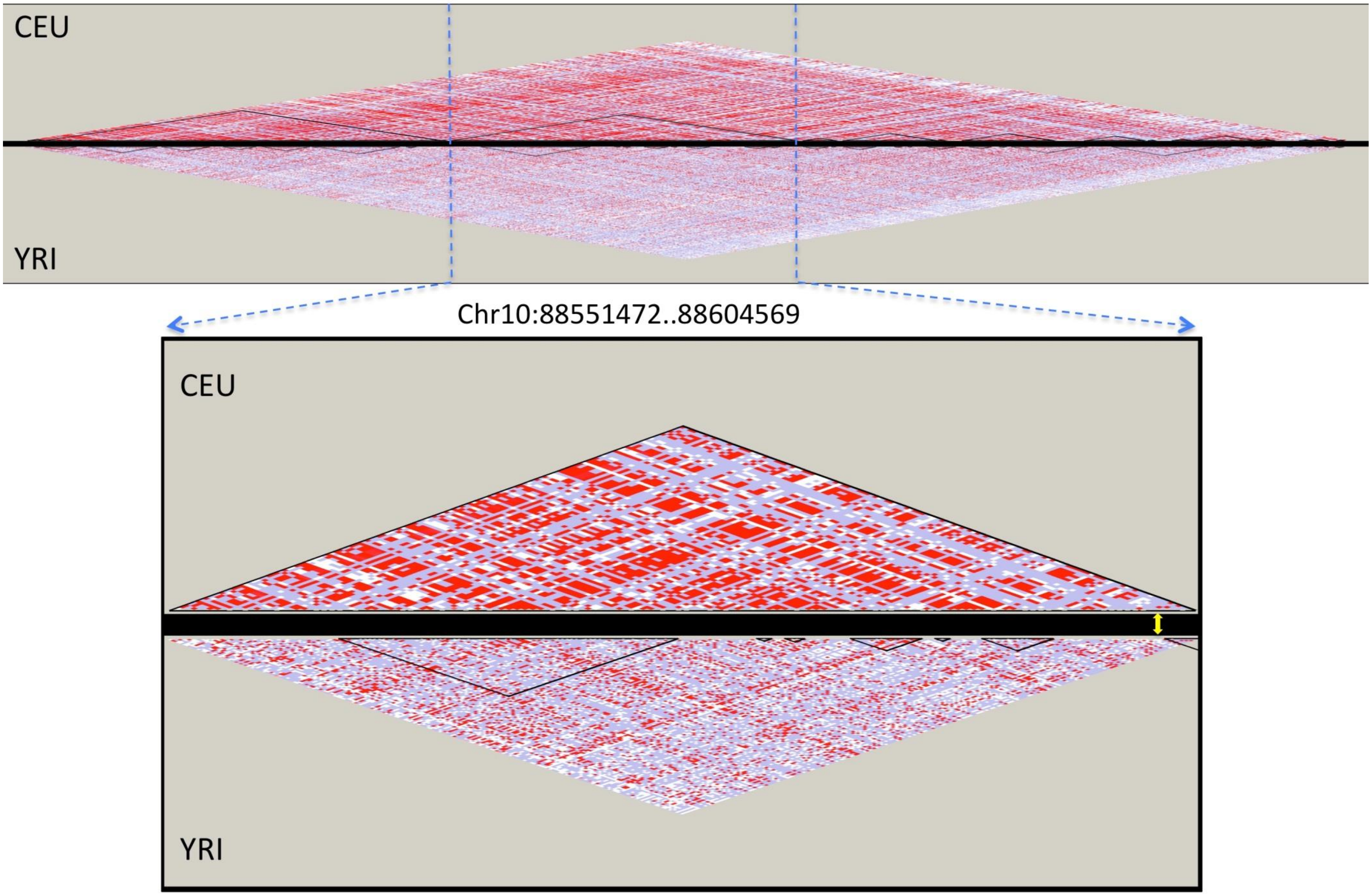
CEU
YRI
Chr10:88551472..88604569
CEU
YRI
